# The Feasibility, Proficiency, and Mastery Learning Curves in 635 Robotic Pancreatoduodenectomies Following a Multicenter Training Program: “Standing on the Shoulders of Giants”

**DOI:** 10.1097/SLA.0000000000005928

**Published:** 2023-06-08

**Authors:** Maurice J.W. Zwart, Bram van den Broek, Nine de Graaf, José A. Suurmeijer, Simone Augustinus, Wouter W. te Riele, Hjalmar C. van Santvoort, Jeroen Hagendoorn, Inne H.M. Borel Rinkes, Jacob L. van Dam, Kosei Takagi, Khé T.C. Tran, Jennifer Schreinemakers, George van der Schelling, Jan H. Wijsman, Roeland F. de Wilde, Sebastiaan Festen, Freek Daams, Misha D. Luyer, Ignace H.J.T. de Hingh, Jan S.D. Mieog, Bert A. Bonsing, Daan J. Lips, Mohamed Abu Hilal, Olivier R. Busch, Olivier Saint-Marc, Herbert J. Zeh, Amer H. Zureikat, Melissa E. Hogg, Bas G. Koerkamp, Isaac Q. Molenaar, Marc G. Besselink

**Affiliations:** * Department of Surgery, Amsterdam UMC, location University of Amsterdam, Amsterdam, the Netherlands; † Cancer Center Amsterdam, Amsterdam, the Netherlands; ‡ Department of Surgery, Erasmus MC Cancer Institute, Rotterdam, the Netherlands; § Department of Surgery, Fondazione Poliambulanza Institute, Brescia, Italy; ∥ Department of Surgery, Regional Academic Cancer Center Utrecht, UMC Utrecht Cancer Center & St Antonius Hospital Nieuwegein, Utrecht University, Utrecht, the Netherlands; ¶ Department of Surgery, Amphia Medical Center, Breda, the Netherlands; # Department of Surgery, OLVG, Amsterdam, the Netherlands; ** Department of Surgery, Catharina Hospital, Eindhoven, the Netherlands; †† Department of Surgery, Leiden University Medical Center, Leiden, the Netherlands; ‡‡ Department of Surgery, Twente Medical Spectrum, Enschede, the Netherlands; §§ Department of Surgery, Southampton University Hospital NHS Foundation Trust, Southampton, UK; ∥∥ Department of Surgery, Orleans Regional Medical Center, Orleans, France; ¶¶ Department of Surgery, University of Texas, Southwestern, Dallas, TX; ## Department of Surgery, University of Pittsburgh Medical Center, Pittsburgh, PA; *** Department of Surgery, Northshore University HealthSystem, Chicago, IL

**Keywords:** clinical outcomes, learning curve, robotic pancreatoduodenectomy, robotic surgery

## Abstract

**Objective:**

To assess the feasibility, proficiency, and mastery learning curves for robotic pancreatoduodenectomy (RPD) in “second-generation” RPD centers following a multicenter training program adhering to the IDEAL framework.

**Background:**

The long learning curves for RPD reported from “pioneering” expert centers may discourage centers interested in starting an RPD program. However, the feasibility, proficiency, and mastery learning curves may be shorter in “second-generation” centers that participated in dedicated RPD training programs, although data are lacking. We report on the learning curves for RPD in “second-generation” centers trained in a dedicated nationwide program.

**Methods:**

Post hoc analysis of all consecutive patients undergoing RPD in 7 centers that participated in the LAELAPS-3 training program, each with a minimum annual volume of 50 pancreatoduodenectomies, using the mandatory Dutch Pancreatic Cancer Audit (March 2016–December 2021). Cumulative sum analysis determined cutoffs for the 3 learning curves: operative time for the feasibility (1) risk-adjusted major complication (Clavien-Dindo grade ≥III) for the proficiency, (2) and textbook outcome for the mastery, (3) learning curve. Outcomes before and after the cutoffs were compared for the proficiency and mastery learning curves. A survey was used to assess changes in practice and the most valued “lessons learned.”

**Results:**

Overall, 635 RPD were performed by 17 trained surgeons, with a conversion rate of 6.6% (n=42). The median annual volume of RPD per center was 22.5±6.8. From 2016 to 2021, the nationwide annual use of RPD increased from 0% to 23% whereas the use of laparoscopic pancreatoduodenectomy decreased from 15% to 0%. The rate of major complications was 36.9% (n=234), surgical site infection 6.3% (n=40), postoperative pancreatic fistula (grade B/C) 26.9% (n=171), and 30-day/in-hospital mortality 3.5% (n=22). Cutoffs for the feasibility, proficiency, and mastery learning curves were reached at 15, 62, and 84 RPD. Major morbidity and 30-day/in-hospital mortality did not differ significantly before and after the cutoffs for the proficiency and mastery learning curves. Previous experience in laparoscopic pancreatoduodenectomy shortened the feasibility (−12 RPDs, −44%), proficiency (−32 RPDs, −34%), and mastery phase learning curve (−34 RPDs, −23%), but did not improve clinical outcome.

**Conclusions:**

The feasibility, proficiency, and mastery learning curves for RPD at 15, 62, and 84 procedures in “second-generation” centers after a multicenter training program were considerably shorter than previously reported from “pioneering” expert centers. The learning curve cutoffs and prior laparoscopic experience did not impact major morbidity and mortality. These findings demonstrate the safety and value of a nationwide training program for RPD in centers with sufficient volume.

Several “pioneering” high-volume centers have described excellent outcomes for robotic pancreatoduodenectomy (RPD).^1–8^ Some centers reported a shorter hospital stay and even a lower risk of postoperative pancreatic fistula (POPF) after RPD, as compared with open pancreatoduodenectomy (OPD).^9,10^ Based on these reports there is a growing interest in high-volume centers to start with RPD. However, concerns exist regarding the long learning curves reported from these “pioneering” expert centers. The University of Pittsburgh Medical Center (UPMC) group reported a feasibility learning curve of 80 RPDs and mastery obtained at 240 RPDs.^7,11^

The recent international evidence-based Miami guidelines strongly advise participation in a structured training program for minimally invasive PD.^12^ In 2020, the Dutch Pancreatic Cancer Group (DPCG) reported on the LAELAPS-3 training (2016–2019) for RPD which was developed together with 3 surgeons from the UPMC group.^13–15^ When the early results were reported from this training program only 4 centers had completed >20 RPDs. Recently, 2 other types of learning curve have been reported.^16,17^ The “proficiency” learning curve using risk-adjusted complications, and the “mastery” learning curve, using risk-adjusted textbook outcomes. The latter may require experience up to 30 to 80 and 160 to 250 RPD procedures based on data from a systematic review by Müller and colleagues and single-center reports.^7,17–19^ Furthermore, it is unclear to what extent previous experience with laparoscopic pancreatoduodenectomy (LPD) may shorten these learning curves of robotic pancreatic procedures.

Data on these 3 learning curves from “second-generation” centers which followed a dedicated RPD training program are lacking. This is relevant to inform new centers that may be discouraged by the long learning curves reported by the “pioneering” centers. We hypothesize that the learning curves for RPD are shorter in trained “second-generation” centers as compared with “pioneering” centers. Now, 2 years after our initial report,^20^ we report on the feasibility, proficiency, and mastery learning curves for RPD including the clinical impact of these learning curves and previous experience with LPD.

## METHODS

### Patients and Design

This is a post hoc analysis of outcomes of all consecutive RPD during and following the Dutch LAELAPS-3 multicenter training program in RPD, including the first RPD in every participating center. The study was designed and performed in collaboration with the UPMC group (M.E.H., H.J.Z., and A.H.Z.).^13^ Data were retrieved from the mandatory Dutch Pancreatic Cancer Audit (March 2016–December 2021).^21^ Data on RPD procedures from one center which did not participate in the nationwide training program were excluded. In addition, nationwide trends on the use of RPD and LPD were obtained via the Dutch Pancreatic Cancer Audit to assess practice shifts over time. This study followed the guidelines for Strengthening the Reporting of Observational Studies in Epidemiology (STROBE).^22^ The scientific committee of the Dutch Pancreatic Cancer Group approved this project^23^ and the medical ethics review committee of Amsterdam UMC waived the need for informed consent due to the observational nature of this study (W17_129#17.149). This study was registered at the International Clinical Trials Registry Platform (identification number: NL8073).

### Three Learning Curves

The feasibility learning curve was based on operative time. The proficiency learning curve was based on risk-adjusted major complications (Clavien-Dindo grade 3 or higher)^24^. The mastery learning curve was based on textbook outcome defined according to Müller et al^17^: hospital stay shorter inside the 75th percentile, (ie, <20 days); no mortality; no complication requiring (medium) intensive care unit admission; and no reoperation.^17^

### Outcomes

Surgical and postoperative outcomes were assessed in both phases (ie, before vs after the cutoff) of the 3 learning curves of RPD. Herein, the safety outcomes were conversion, major complications, and textbook outcomes. Other outcomes included blood loss, delayed gastric emptying, wound infection, POPF, postpancreatectomy hemorrhage, bile leakage, chyle leakage, readmission, in-hospital/30-day mortality, length of stay, and reoperations.

### Definitions

Conversion was defined as an urgent or nonurgent switch to open laparotomy to complete the procedure, other than specimen extraction.^25^ An extracorporeal gastric anastomosis performed through the extraction site was not considered a conversion. Operative time was defined as the time between the first incision and the final closure of incisions. Postoperative complications were classified using the Clavien-Dindo classification of surgical complications with grade 3 or higher defined as major morbidity.^24^ The definitions of the International Study Group on Pancreatic Surgery (ISGPS) were used to score POPF, delayed gastric emptying, postpancreatectomy hemorrhage, bile leakage, and chyle leakage.^26–30^ Only the clinically relevant grade B and C complications were included. Wound infection (surgical site infection) required at least opening, flushing, and covering of the wound with gauze.^21,31^ Resection margins were categorized according to the Royal College of Pathologists definition and classified into R0 (distance margin to tumor ≥1 mm), R1 (distance margin to tumor <1 mm), and R2 (macroscopically positive margin).^32^ Complications requiring readmission and/or reoperation were recorded up to 30 days postoperatively. The Miami guideline volume cutoff of 20 RPDs/year was assessed per center for each individual full calendar year. Additional resection was defined according to the ISGPS.^33^ Vascular resection was classified according to resections of the portomesenteric, splenic, or inferior mesenteric vein. Arterial resections were classified according to resection of the hepatic artery, superior mesenteric artery, or celiac trunk. Risk categories for pancreatic anastomosis were defined according to the ISGPS classification: (A) not-soft (hard) texture and main pancreatic duct (MPD) >3 mm; (B) hard texture and MPD ≤3 mm; (C) soft texture and MPD >3 mm; and (D) soft texture and MPD ≤3 mm.^34^ The pancreatoduodenectomy difficulty score was defined according to Büchler et al^35^: (I) no additional resection; (II) venous resection; (III) additional resection; (IV) arterial resection.^34^

### Survey

A short survey was developed using Google Forms Survey (Google) and was disseminated by email to all surgeons performing RPD (Supplemental Material 1, Supplemental Digital Content 1, http://links.lww.com/SLA/E628). The survey included questions with regards to surgical experience, case selection, training, surgical experience, case selection for robotic pancreatectomy, and lessons learned during the training program and thereafter.

### Data Collection

Data were prospectively collected in the DPCA database during the hospital stay and after discharge up to 30 days postoperatively. Collected baseline characteristics were sex, age (years), body mass index (BMI, kg/m^2^), comorbidity and medical history, American Society of Anesthesiologists physical status, pancreatic duct diameter (mm), and pancreatic texture (soft or firm). Collected outcomes were conversion, operative time (minutes), measured intraoperative blood loss (mL, combining blood in the suction canister and in gauzes), histopathologic diagnosis, tumor size (mm), resection margins, and lymph node retrieval. Collected postoperative outcomes were POPF, bile leakage, delayed gastric emptying, postpancreatectomy hemorrhage, chyle leakage, wound infections, intensive care unit admission, complications (Clavien-Dindo classification), length of hospital stay (days), readmission, neoadjuvant chemo(radio)therapy, in-hospital mortality, and 30-day mortality.

### Statistical Analyses

Data were analyzed using IBM SPSS Statistics for Windows, version 28 (IBM Corp.). Student *t*, Mann Whitney *U*, χ^2^, or Fisher exact tests were used as suitable. Categorical data were presented as proportions, continuous data were presented as either mean and SD or median and interquartile range (IQR) as applicable. α was set at a *P* value <0.05, and all analyses were 2-sided. Missing data were resolved by multiple imputations wherever appropriate.

#### Subgroup Analyses

Subgroup analyses compared (a) outcomes of centers in the years wherein the Miami guidelines volume advice^12^ was met versus others, and (b) surgical teams with and without LPD experience.

#### Learning Curve Cumulative Sum (CUSUM) Analysis

The learning effects were assessed with CUSUM analyses. First, patients were ranked consecutively according to the date of their procedure and the difference of the data to the mean per center was calculated per case. Hereafter, data was aggregated for all centers, and hereafter a CUSUM data was presented on the *y* axis with the ranked consecutive case numbers presented on the *x* axis. The magnitude by which the line ascends or descends is determined by the difference between the observed and expected outcome. For example, the line ascends when operative time in that case was above average for that center by an amount relative to the SD, and for a case with operative time below average, the line descends. The top of the CUSUM graph thus represented the total operative expressed in SDs above average up to that case. When interpreting the CUSUM graph, “slope” is the informative part, wherein an uphill slope indicates an outcome above average and a downhill slope indicates an outcome below average for that consecutive case number. The turning point of curvature indicates the point at which the centers transition from one phase to another and overcomes the specific learning curve (indicated by #n=turning point case number). The turning point determined cutoffs for the feasibility and proficiency learning curves were then used to compare operative outcomes. CUSUM analyses assessed the feasibility of learning curves comprised of operative time. RA-CUSUM analysis assessed the proficiency learning curve (major complications) and mastery learning curve (textbook outcome). For risk-adjusting, a regression analysis was performed with variables identified from the I-MIPS RPD cohort (age, BMI, American Society of Anesthesiologists; center),^36^ pancreatic anastomosis classification, and pancreatoduodenectomy difficulty score.

## RESULTS

### Implementation

**FIGURE 1:**
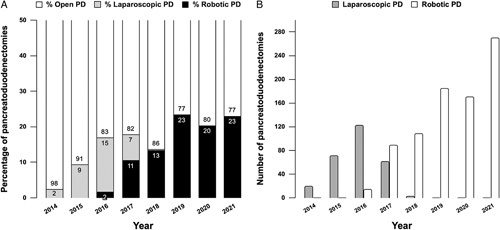
Nationwide use of LPD and RPD in the Netherlands. A, The proportion of minimally invasive pancreatoduodenectomies per year. Gray indicates the LPDs, black indicates the RPDs. B, The absolute number of minimally invasive pancreatoduodenectomies per year. Gray indicates the LPDs, white indicates the RPDs.

Between 2016 and 2021, the nationwide use of RPD increased from 0% to 23% (269/1166). In 2016, 3 centers started with RPD, in 2017 1 additional center followed, and in 2018 3 more centers. For more details on implementation, see Figure 1. In the study period, the use of LPD decreased from 15% to 0%.

### Patient Demographics

Overall, 635 patients undergoing RPD were included from 7 trained centers (mean n=91 per center, range: 47–166). The Miami annual volume recommendation of 20 RPDs was met in 16 (62%) of 26 study years (cumulative full years). In the final study year, all but one center met the Miami annual volume advice. This one center did not meet this criterium in any year but performed at least 10 annual RPDs and performed at least 20 robotic pancreatic resections annually and other robotic procedures. Median patient age was 69 (IQR: 61–75) years and the mean BMI was 25 (IQR: 23–28) kg/m^2^. Patient demographics are presented in Table 1. The ISGPS A–D risk pancreatic anastomosis classification was available for 499 patients: A, n=108 (21.6%); B, n=64 (12.9%); C, n=93 (18.7%); D, n=234 (46.9%). On preoperative imaging, vascular involvement was observed in 81 patients (12.4%) (90–180 degrees in 5.4% (n=35) and <90 degrees in 7.0% (n=46) which led to a vascular resection in 47/81 58% (performed robotically in 37 patients).

**TABLE 1:** Baseline Characteristics

	N=635
Patient characteristics	
Age [median (IQR)] (yr)	69 (61–75)
Age≥75 yr	139 (21.9)
BMI [median (IQR)] (kg/m^2^)	25 (23–28)
BMI ≥30 kg/m^2^	81 (12.8)
Male	354 (55.7)
Comorbidity and medical history, any	396 (62.4)
Diabetes	129 (20.3)
Pulmonary disease	93 (14.6)
Cardiovascular disease	90 (14.2)
Peripheral vascular disease	65 (10.2)
Oncologic disease <5 yr prior	51 (8.0)
Pancreatitis	51 (8.0)
Cerebrovascular attack	40 (6.3)
Kidney disease	28 (4.4)
Gastric ulcer disease	14 (2.2)
Liver disease	12 (1.9)
ASA physical status	
I and II	431 (67.9)
III and IV	204 (32.1)
Neoadjuvant chemo(radio)therapy	46 (7.2)
Pancreatic classification, available in n=499	
(A) Not-soft (hard) texture and MPD >3 mm	108 (21.6)
(B) Not-soft (hard) texture and MPD ≤3 mm	64 (12.9)
(C) Soft texture and MPD >3 mm	93 (18.7)
(D) Soft texture and MPD ≤3** **mm	234 (46.9)
Disease characteristics	
Vascular involvement	81 (12.4)
Malignant disease	426 (67.1)
Pancreatic cancer	165 (26.0)
Distal cholangiocarcinoma	88 (13.9)
Ampullary cancer	110 (17.3)
Other	63 (9.9)
Premalignant/benign disease	209 (32.9)
Intraductal papillary mucinous neoplasm	88 (13.9)
Adenoma	27 (4.3)
Autoimmune or IgG4-related disease	15 (2.4)
Chronic pancreatitis	20 (3.1)
Other benign	59 (9.3)

ASA indicates American Society of Anesthesiologists; Ig, immunoglobulin.

### Surgeon and Center Demographics

The number of surgeons performing RPD per center ranged from 1 to 4. Six of the 7 centers performed RPD with 2 senior surgeons, consisting of a “console surgeon” and a “table side surgeon.” The remaining center used a one-surgeon approach with 2 dedicated assistants. In 4 of the 6 centers, surgeons switched roles after the resection phase. Median surgical experience was 19 (11–24) years, with the current focus (93%) on pancreatic surgery. The median minimally invasive experience was 15 (10–24) years. At the end of the study, the median individual surgeon experience was 45 (30–60) RPDs.

### Intraoperative Outcome and Pathology

The median operative time was 395 (341–465) minutes and the median blood loss was 200 (100–450) mL. Conversion to an open approach was performed in 42 patients (6.6%). Median tumor size was 25 (17–35) mm, of which 67.1% were malignant. Pancreatic cancer was the final diagnosis in 165 patients (26.0%). In patients with malignant disease, the median lymph node harvest was 15 (12–19), free resection margins rate was 80.8% (n=426), and the R0 resection rate (≥1 mm definition) was 70.9% (n=302). Intraoperative outcomes are presented in Table 2.

**TABLE 2:** Operative Outcomes

Operative outcome	Total N=635 [n (%)]
Pylorus resecting PD	488 (76.9)
Pylorus preserving PD	147 (23.1)
Total operative time [median (IQR)] (min)	395 (341−465)
Operative time <360 min	213 (33.5)
Blood loss [median (IQR)] (mL)	200 (100–450)
Blood loss >500 mL	118 (18.6)
Blood loss >1000 mL	34 (5.4)
Conversion	42 (6.6)
Vascular resection	47 (7.4)
Venous resection	43 (6.8)
Wedge resection	32 (5.1)
Segmental venous resection	11 (1.7)
Arterial resection	4 (0.6)
Additional resection	20 (3.6)
Extracorporeal gastric anastomosis	147 (23.1)

### Postoperative Outcome

The rate of major postoperative complications was 36.9% (n=234), of which 61 (9.6%) required reoperation. The rate of POPF was 26.9% (n=171), bile leakage 8.0% (n=51), chyle leakage 2.7% (n=17), postpancreatectomy hemorrhage 12.1% (n=77), delayed gastric emptying 23.5% (n=149), and wound infection 6.5% (n=41). In patients with pancreatic ductal adenocarcinoma (PDAC) as a histologic diagnosis compared with other diagnoses, the rate of POPF was 9.7% (16/165) versus 33.0% (155/470) *P*<0.001, respectively. The median length of hospital stay was 11 (7–19) days. The 30-day readmission rate was 22.8% (n=145). The in-hospital/30-day mortality rate was 3.5% (n=22), all of which as a cause of major morbidity (failure to rescue rate 9.4%).

### The Feasibility, Proficiency, and Mastery Learning Curves

#### Feasibility

**FIGURE 2:**
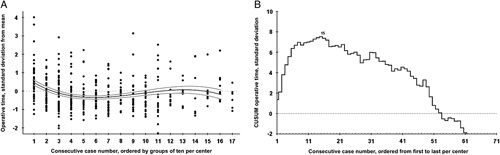
Feasibility learning curve of operative time for RPD. A and B, The *x* axis indicates groups of 10 consecutive RPDs ranked from first to last per center, and the *y* axis indicates the operative time in SDs from the mean. B, The black line indicates CUSUM analysis of operative time. The label (n=15) indicates the first top turning point of the learning curve, where after, a continuous downward slope occurs.

The CUSUM analysis of operative time revealed a cutoff for the feasibility learning curve at 15 RPD procedures. The rates of conversion, major complications, and the textbook outcome did not differ significantly between the 2 phases of the feasibility learning curve. Operative time decreased from 437 to 386 minutes (*P*<0.001) and operative time below 360 minutes was attained in 18.9% versus 29.7% of patients (*P*=0.005). Length of initial hospital stay decreased [median: 13 (9–21)–11 (7–19) days, *P*=0.029] and the rate of hospital stay <7 days, increased (6.7%, n=7 vs 17.2%, n=91, *P*=0.002). For more details, see Figure 2. For the outcome analysis, see Supplementary Material 2 (Supplemental Digital Content 2, http://links.lww.com/SLA/E629).

#### Proficiency

**FIGURE 3:**
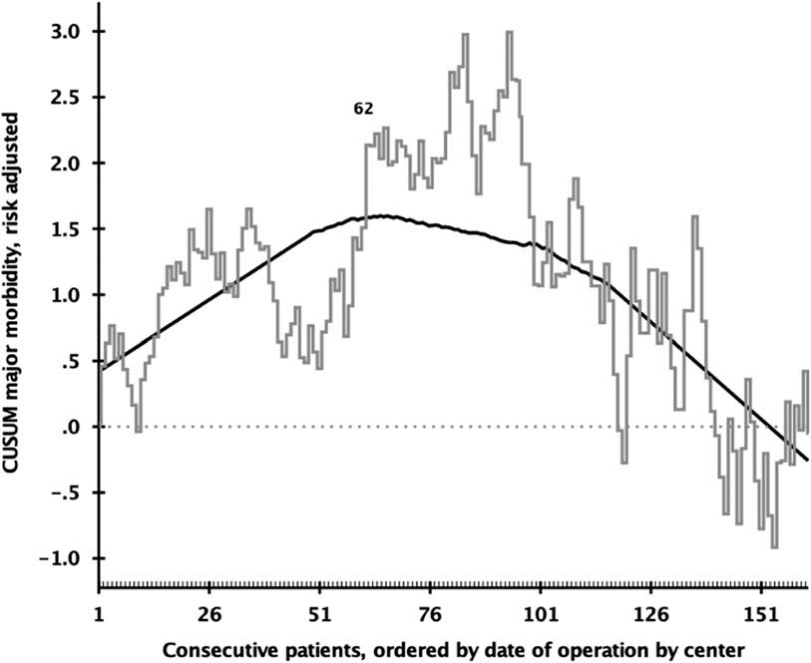
Proficiency learning curve of risk-adjusted major morbidity for RPD. The *x* axis indicates groups of consecutive RPDs ranked from first to last per center, and the black line indicates the risk-adjusted CUSUM analysis of major morbidity. The first label (n=62) indicates the first top turning point of the learning curve, where after, a continuous downward slope occurs.

Risk-adjusted CUSUM analysis of major morbidity revealed a cutoff for the proficiency learning curve at 62 RPD procedures (Fig. 3). The rates of conversion (7.8% vs 4.4%, *P*=0.069), major complications (35.4% vs 39.6%, *P*=0.295), and textbook outcome (70.2% vs 64.9%, *P=*0.165) did not differ significantly between the 2 phases of the proficiency learning curve. The rates of delayed gastric emptying (25.7%–16.0%, *P*<0.001), wound infections (8.8%–1.8%, *P*<0.001), and reoperations (12.0%–4.9%, *P*=0.004) all decreased, as did median blood loss [200 (100–500) mL–200 (136–400) mL, *P*=0.004]. The rates of postpancreatectomy hemorrhage, bile leakage, chyle leakage, readmission, mortality, and length of stay remained stable. Although the rate of POPF increased (from 23.2% to 33.8%), the rate of grade C pancreatic fistula decreased (from 2.4% to 0.4%), *P*=0.004 (Table 3).

**TABLE 3:** Outcomes in Learning Phases

	Before proficiency cutoff ≤62 RPD (N=410) [n (%)]	After proficiency cutoff >62 RPD (N=225) [n (%)]	*P*	Before mastery cutoff ≤84 RPD (N=480) [n (%)]	After mastery cutoff >84 RPD (N=155) [n (%)]	*P*
Safety outcomes						
Conversion	32 (7.8)	10 (4.4)	0.069	33 (6.9)	9 (6.2)	0.400
Clavien-Dindo complication ≥III	145 (35.4)	89 (39.6)	0.295	173 (36.0)	61 (39.4)	0.543
Requiring catheter drainage	126 (30.7)	84 (37.3)	0.091	169 (33.1)	41 (32.8)	0.517
Reoperation	50 (12.2)	11 (4.9)	0.004	53 (11.0)	8 (5.2)	0.018
Unplanned intensive care unit admission	55 (13.4)	16 (7.1)	0.010	56 (11.7)	15 (9.7)	0.494
Textbook outcome	288 (70.2)	146 (64.9)	0.165	334 (69.6)	100 (64.5)	0.238
Type I^34^ only (no additional resection)	250/346 (72.3)	145/216 (67.1)	0.053	293/410 (71.5)	92/145 (62.8)	0.072
Other outcomes						
Length of initial stay [median (IQR)] (d)	11 (7–18)	11 (7–19)	0.654	11 (7–19)	11 (7–21)	0.488
Initial hospital stay <7 d	66 (16.1)	33 (14.7)	0.371	79 (16.5)	19 (12.3)	0.270
Readmission	95 (23.1)	50 (22.2)	0.823	104 (21.7)	41 (26.4)	0.216
In-hospital/30-d mortality	14 (3.4)	8 (3.5)	0.629	15 (3.1)	7 (4.5)	0.410
POPF (B/C)	95 (23.2)	76 (33.8)	0.004	131 (24.2)	40 (35.5)	<0.001
Of which grade C	10 (2.4)	1 (0.4)		11 (2.3)	0 (0.0)	
Grade B/C in class A and B (soft)	61/250 (24.4)	57/156 (36.5)	0.006	75/296 (25.3)	43/110 (39.1)	0.007
Grade B/C in class C and D (hard)	13/71 (18.3)	7/22 (31.8)	0.178	17/78 (21.8)	3/15 (20.0)	0.591
Bile leakage (B/C)	31 (7.6)	20 (8.9)	0.308	39 (8.1)	12 (7.7)	0.517
Delayed gastric emptying (B/C)	113 (27.6)	36 (16.0)	0.001	124 (25.8)	25 (16.1)	0.008
Postpancreatectomy hemorrhage (B/C)	53 (13.0)	24 (10.6)	0.228	56 (11.7)	21 (13.5)	0.400
Chyle leakage (B/C)	9 (2.2)	8 (3.6)	0.540	11 (2.3)	6 (3.9)	0.752
Wound infection^*^	36 (8.8)	4 (1.8)	<0.001	39 (8.1)	1 (0.6)	<0.001
Oncologic outcome						
Tumor size [median (IQR)] (mm)	25 (18–33)	25 (15–35)	0.584	25 (18–35)	25 (15–35)	0.654
Lymph node harvest^†^ [n (IQR)]	15 (12–19)	14 (11–17)	0.024	15 (12–19)	14 (10–17)	0.013
R0 resection^†^	216/294 (73.5)	97/132 (73.4)	0.377	253/345 (73.3)	60/81 (74.1)	0.813
R0 resection in PDAC	60/110 (54.5)	31/48 (64.6)	0.132	69/127 (54.3)	22/31 (71.0)	0.230

* Wound infection (surgical site infection) required at least opening, flushing and covering of the wound with gauze.

† In patients with malignant disease.

#### Mastery

**FIGURE 4:**
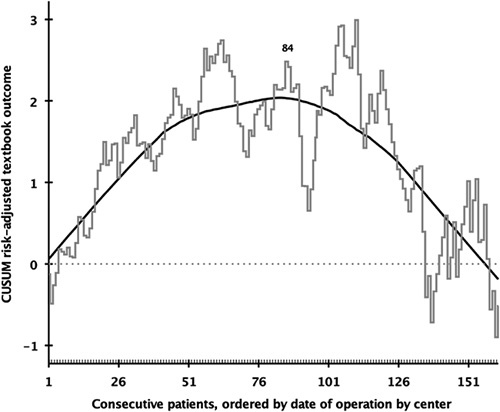
Mastery learning curve of risk-adjusted textbook outcome in RPD. The *x* axis indicates groups of consecutive RPDs ranked from first to last per center, and the black line indicates the risk-adjusted CUSUM analysis of textbook outcome. The first label (n=84) indicates the first top turning point of the learning curve, where after, a continuous downward slope occurs.

Risk-adjusted CUSUM analysis of textbook outcome revealed a cutoff for the mastery learning curve at 84 RPD procedures (Fig. 4). The rates of conversion (6.9% vs 6.2%, *P*=0.400), major complications (36.0%–39.4%, *P*=0.543), and textbook outcome (70.0%–67.0%, *P*=0.238) did not differ significantly between the 2 phases of the mastery learning curve. The rates of delayed gastric emptying (25.8%–16.1%, *P*=0.008) and wound infections (8.1% to 0.6%, *P*<0.001) decreased. The rate of reoperations was significantly less after the turning point: 11.0%–5.2%, *P*=0.018. Whereas the rate of clinically relevant POPF did not change, the rate of fistula grade C decreased (2.3%–0%, *P*<0.001). The rates of postpancreatectomy hemorrhage, bile leakage, chyle leakage, readmission, mortality, and length of stay remained stable between the 2 phases. For more details, see Table 3.

### Survey on the Impact of Training and Experience

The survey results are presented in Supplemental Material 3 (Supplemental Digital Content 3, http://links.lww.com/SLA/E630). Most surgeons would strongly recommend LAELAPS-3 to be repeated in other countries (median strength 9 [8.5–10]). The most mentioned added value of LAELAPS-3 was the structured approach combined with written material, biotissue with feedback, videos, and on-site proctoring all focusing on the same operative approach. The 3 most mentioned “valuable technical aspects” taken from LAELAPS-3 were: (1) structured methods for the anastomosis; (2) strict adherence to procedural steps; (3) feedback by peers. Main changes made to the procedure by participating surgeons after full training were: (1) interrupted sutures for the hepaticojejunostomy anastomosis regardless of bile duct size and wall thickness in 5 centers aiming to reduce biliary leak rates; (2) mobilization of the first jejunal loop from the right side in 4 centers, aiming to reduce the need for “re-docking” and instrument collision; (3) gastric anastomosis through the extraction site in 2 centers, as an attempt to mitigate delayed gastric emptying and reduce operating time.

### Impact of Laparoscopic Experience

In 3 centers with previous experience in LPD, the feasibility learning curve was shorter, 10 versus 22 RPDs, the difference −12 RPDs (−44%). Similarly, the median operative time was 88 minutes shorter in these centers [342 (302–385) vs 430 (380–508) minutes, *P*<0.001]. The prior experience did not reduce the rate of major complications (39.4% vs 35.5%, *P*=0.337), textbook outcome (76.7% vs 68.9%, *P*=0.081), and in-hospital/30-day mortality (3.6% vs 3.4%, *P*=0.876). In centers with experience in LPD, the proficiency learning curve was also shorter, 62 versus 94 RPDs, difference −32 RPDs (−34%). Finally, in these centers, the mastery learning curve was also shorter. This reduction was seen in 2 phases: phase I: 65 versus 21 RPDs; phase II: 110 versus 87 RPDs; average difference: −34 RPDs (−23%) (Supplemental Material 4, Supplemental Digital Content 4, http://links.lww.com/SLA/E631).

### Impact of Volume

In centers meeting the Miami guidelines volume advice,^12^ the rate of conversion (5.3% vs 11.3%, *P*=0.010) was less as compared with other centers. Operative time (415 vs 408 minutes, *P*=0.982), blood loss (368 vs 449 mL, *P*=0.252), major morbidity (32.6% vs 38.1%, *P*=0.238), and 30-day/in-hospital mortality (2.8% vs 5.7%, *P*=0.085) did not differ significantly between centers meeting the Miami guideline volume advice and those who did not (Supplemental Material 5, Supplemental Digital Content 5, http://links.lww.com/SLA/E632).

## DISCUSSION

This first multicenter study reporting on the feasibility, proficiency, and mastery learning curves in 7 “second-generation” centers, specifically trained in RPD, found learning curve cutoffs at 15, 62, and 84 RPDs in 635 RPD procedures. These cutoffs did not affect the conversion rate and had no negative impact on major complications, textbook outcome, and in-hospital/30-day mortality. Previous experience in LPD was associated with shorter learning curves (range −23% to −44%). Altogether, this experience shows markedly shorter learning curves as previously reported from RPD “pioneering” centers.

Previous studies have reported on the feasibility^2,11,37–44^, proficiency^7,11,36^, and mastery^7,36^ learning curves of RPD. For the feasibility learning curve (ie, operative time), a meta-analysis reported an average cutoff of 25 RPD.^17^ Thus, the cutoff of 15 RPDs in the present study is considerably earlier, including the cutoff of 22 RPDs in centers without LPD experience. Second, a systematic review found a proficiency learning curve (ie, risk-adjusted major complications decreased by 46%, of which POPF decreased by 48%) cutoff of 100 RPDs.^17,36,40^ Again, the cutoff of 62 RPD in this present study is considerably earlier and showed no negative impact on outcome in patients who were included before the learning curve cutoff. Third, this is the first multicenter study to report a CUSUM analysis learning curve for the mastery learning curve (ie, textbook outcome).^17^ Zureikat et al^7^ reported that operating times plateaued after 240 procedures and considered this the “mastery” cutoff. Again, the cutoff of 84 RPDs in the present study was considerably earlier.

The largest single-center series of 500 RPD procedures in the Western world is from the UPMC group, which were the proctors of the LAELAPS-3 training program.^7^ We compared outcomes from our multicenter study (7 centers) to their monocenter study, the present results regarding operative time, blood loss, and conversion were similar. However, the term “proficiency” in the current study should be nuanced by the UPMC data, with lower rates of POPF (7.8% vs 27%), major morbidity (24.8% vs 37%), and 30-day mortality (1.4% vs 3.5%).^7^ For more details, see Supplemental Material 6 (Supplemental Digital Content 6, http://links.lww.com/SLA/E633). As the RPD data are comparable to that of the OPD in the Netherlands,^45^ these data also demonstrate that outcomes in the Dutch centers may further improve with increasing experience. We will continue to monitoring our outcomes, especially since only a limited number of centers had performed >100 RPDs in the current cohort. Some differences may also exist in patient selection, volume, and postoperative management. For example, a difference in risk factors for POPF exists between the UPMC study (46% PDAC) and the current study (26% PDAC).

We will also continue to collaborate with the 3 US-based proctors and other international surgeons to learn from each other with the aim to improve the outcomes of RPD in the Netherlands. Especially regarding the 8% biliary leak rate, 26.9% POPF rate, and 3.5% in-hospital mortality rate. Although part of these results could be related to patient selection for RPD it will be interesting to see whether these outcomes improve with increasing experience. We aim to further investigate these hypotheses when all centers have performed >100 RPDs and compare these outcomes to international expert centers. Some evidence points to patient selection as a partial explanation for the higher biliary leak rate. Patients for minimally invasive PD in this series were typically selected on the basis of the absence of vascular involvement which is often accompanied by a nondilated bile duct and pancreatic duct and hence higher leak rates. Notably, in the 4 available randomized controlled trials on LPD versus OPD, the bile leak rates were on average 7.4% and 7.6%, respectively.^46–48^

Clearly, the shorter learning curves in the 7 centers were a tribute to and result of the combination of close collaboration with the UPMC group in the structured LAELAPS-3 training program and the previous experience of intermediate-volume surgeons in our group. Previous training programs (LAELAPS-1 and LAELAPS-2) were performed in the Netherlands for laparoscopic distal pancreatectomy and LPD.^49,50^ The seemingly short feasibility learning curve (ie, 15 RPDs), should be viewed in the light of the previous training of the participating surgeons and structured training program including group reflection meetings with ongoing proctoring/mentoring during the proficiency and mastery learning curve. This is reflected by the later turning points in the learning curves for conversion (37 RPDs) and blood loss (43 RPDs). Furthermore, volume criteria might apply, therefore, the European Consortium on Minimally Invasive Pancreatic Surgery is performing the LEARNBOT training program for RPD, endorsed by the European-African Hepato-Pancreato-Biliary Association (E-AHPBA), again only in centers performing at least 50 pancreatoduodenectomies per year, either in dual-approach or single-surgeon approach. Although the dual-surgeon approach demonstrated the longest learning curve in the current study, the relevance of this is unclear as only one center used the single-surgeon approach. Naturally, there are other high-value training courses available, such as the “Robotic Whipple Surgery Course” by Giulianotti et al^51^ and the “Surgical training model” by Takagi et al.^52^ The need for training and support during the safe implementation of minimally invasive PD is further reflected by a worldwide survey with over 400 hepato-pancreatico-biliarysurgeons from 50 countries, of which 44% stated they did not perform minimally invasive PD due to the lack of adequate training opportunities.^53^

It is of interest to compare outcomes before and after the cutoffs of the learning curves. If these outcomes do not differ one could conclude that patients had no negative impact based on the learning curve process. For all 3 learning curves, the rate of conversion, major complications, textbook outcome, and in-hospital/30-day mortality did not differ between the periods before and after the cutoffs. However, our learning curve analysis did reveal a significant decrease in reoperation rates, mitigation of POPF severity, bile leakage, postpancreatectomy hemorrhage, wound infections, and delayed gastric emptying. It is therefore likely that the progression of the experience came with a mitigation of the severity of the complications. During the LAELAPS-3 program, the Dutch PORSCH trial investigated whether the implementation of an algorithm for early detection and management of pancreatic fistula may improve outcomes after pancreatic resection and subsequently halved mortality after pancreatic surgery.^54^ This intervention might have increased rate of postoperative fistula, due to increased use of percutaneous drainage, but may also have played a role in the decrease in grade C POPF to 0% after the “mastery” cutoff.

In the current study, the median number of 15 retrieved lymph nodes was similar to the 16 nodes retrieved in a recent Dutch multicenter randomized trial on PD specimen grossing.^55^ Although this median number of 15 lymph nodes is^54^ somewhat below a recent international benchmark cutoff (>16 nodes)^56^ it is much lower than >28 required lymph nodes reported in a recent study.^57^ The rate of R0 resection (>0 mm definition) was 81% in the current study, which conforms to textbook outcomes for pancreatic adenocarcinoma as identified from the National Cancer Database (>77.9%).^58^ However, the R0 resection (>0 mm definition) rate in patients with pancreatic adenocarcinoma of 71% has clear room for improvement. Furthermore, the 74% R0 rate (≥1 mm definition) in malignancy was substantially higher than reported in the earlier experience of the LAELAPS-3 cohort (52.8%).^20^

The survey performed in the present study demonstrated that surgeons largely adhered to the previously determined (relative) contraindications for RPD, namely previous extensive abdominal surgery, history of chronic pancreatitis, central obesity with BMI >35 kg/m^2^ and segmental vascular resections during the early learning curve.^37,59^ Several studies have suggested that robotic vascular resections were safe when performed in highly experienced centers by surgeons who have surpassed the RPD learning curve.^59,60^ In addition, we found that surgeons with experience in LPD had shorter operative time and shorter major morbidity learning curves compared with others, this could have resulted from a transfer of skill van laparoscopy to robotic, for example, trocar placements. Finally, centers with an annual volume of >20 RPD had reduced a conversion rate. Although center volume halved the rate of in-hospital/30-day mortality, this was not statistically significant, possibly because of a type II error. Similar findings were reported from the E-MIPS group for minimally invasive PD.^61,62^

The results of this study should be interpreted in light of some limitations. First, while the data used for this study were collected prospectively, the post hoc nature of the study limits data collection such as for costs and 90-day mortality, which are not available in the Dutch Pancreatic Cancer Audit. Second, this study involved mainly (ie, in 6 of 7 centers) a 2-surgeon approach and may therefore not be generalizable to centers routinely using a one-surgeon approach, for example, there was a median individual experience of 45 RPDs per surgeon, of which 4 surgeons completed the mastery learning curve (>84 RPDs). Third, variations in patient treatment over time might have impacted the learning curves. For example, the increased rate of grade B POPF might be explained by both more drainage interventions as a result of the effort to limit the impact of POPF in the PORSCH trial by earlier detection and proactive drainage.^54^ The major strength of this study is the homogeneous selection criteria for RPD and training provided in the 7 participating centers in combination with the large sample size.

In conclusion, a structured multicenter training program for RPD in “second-generation” centers with sufficient surgical volume demonstrated shorter feasibility, proficiency, and mastery learning curves of 15, 62, and 84 RPDs as compared with previous reports from “pioneering” centers. Prior laparoscopic experience shortened the learning curves but did not reduce major morbidity and mortality. In centers meeting the Miami guidelines volume advice, the conversion rate halved as compared with other centers. Ultimately, randomized studies are needed in high-volume centers with high-volume surgeons who have surpassed the learning curves, to compare outcomes of RPD with the open approach, several of which are currently ongoing, such as the European DIPLOMA-2 trial and the Chinese PLOT trial, or have recently been completed.^63^

## Supplementary Material
