# Supplementary material for: The Feasibility, Proficiency, and Mastery Learning Curves in 635 Robotic Pancreatoduodenectomies Following a Multicenter Training Program: “Standing on the Shoulders of Giants”
Source: Ann Surg. 2023 Jun 8;278(6):e1232–41. doi: 10.1097/SLA.0000000000005928 (PMC10631507; doi:10.1097/SLA.0000000000005928)
Supplement: Supplementary file 1 [file sla-278-e1232-s001.docx]

# Supplemental Digital Content

## Supplemental Material 1. Survey regarding surgical experience, annual case volume, training, case selection, and initial & current standard of care.

### Surgical Experience

For how many years have you been a practicing surgeon?

___ years

Which elective surgical procedures do you perform currently? Multiple options possible.

- Pancreatic
- Liver
- Colorectal
- Upper-GI
- Bariatric

For how many years do you have experience with advanced MI gastrointestinal resections (i.e., beyond appendectomy, cholecystectomy)?

___ years

What type of MI gastrointestinal surgery do you perform? Multiple options possible.

- Pancreatic
- Liver
- Colorectal
- Upper-GI
- Bariatric

Which type of MIS-HPB surgery did you perform for more than 1 year prior to participating in LAELAPS-3? Multiple options possible.

- None
- Laparoscopic distal pancreatectomy
- Robotic distal pancreatectomy
- Laparoscopic pancreatoduodenectomy
- Laparoscopic minor liver resection
- Robotic minor liver resection
- Laparoscopic major liver resection
- Robotic major liver resection

### Case selection

What where your main (relative) contra-indications during the initial 20 RPD cases? Multiple options possible.

- Vascular involvement
- Suspected/confirmed malignancy
- Previous complicated upper abdominal surgery
- Requiring additional colonic/mesocolon resection
- BMI > 30
- BMI > 35
- Chronic pancreatitis
- Recurrent acute pancreatitis
- Post-ERCP pancreatitis (1x)
- Other, namely……

What are your current (relative) contra-indications for RPD?

- Vascular involvement
- Suspected/confirmed malignancy
- Previous complicated upper abdominal surgery
- Requiring additional colonic/mesocolon resection
- BMI > 30
- BMI > 35
- Chronic pancreatitis
- Recurrent acute pancreatitis
- Post-ERCP pancreatitis (1x)
- Other, namely……

### Training

How many times have you been proctored in a RPD procedure?

___ #

What type of pancreatic surgery training did you perform? Multiple options possible.

- Simulation (da Vinci skills simulator)
- Video
  - 1-5 hours
  - 5-10 hours
  - 20-30 hours
  - >40 hours
- Artificial organs
  - Pittsburgh protocol (LAELAPS-3/E-MIPS course/LEARNBOT)
  - Outside Pittsburgh protocol
- Case observation
- Proctoring
  - Off-site
  - On-site
- Fellowship

What was, in your opinion, the main added value of LAELAPS-3?

-----

### Experience

How likely are you to recommend the LAELAPS-3 program to surgeons in other countries if such a program would be available? (0=not at all, 10= absolutely)

____

What is in your view the minimum volume of RPD which a center should perform?

- 50/year
- 40/year
- 30/year
- 20/year
- 10/year

How many RPDs did your center perform on average per year in the past 3 years?

- 50/year
- 40/year
- 30/year
- 20/year
- 10/year

How many surgeons participate in RPD in your center

- 1
- 2
- 3
- 4

With which surgical team do you perform RPD in your center currently?

- 2 surgeons who participated in LAELAPS-3
- 1 surgeon who participated in LAELAPS-3 and 1 other surgeon
- 1 surgeon and 1 fellow
- 1 surgeon and 1 senior resident
- 1 surgeon and 1 scrub nurse

What were the top 3 valuable technical aspects (details) you learned from LAELAPS-3?

Which part of the operation did you change after you were fully trained in LAELAPS-3?
