# Supplementary material for: The Feasibility, Proficiency, and Mastery Learning Curves in 635 Robotic Pancreatoduodenectomies Following a Multicenter Training Program: “Standing on the Shoulders of Giants”
Source: Ann Surg. 2023 Jun 8;278(6):e1232–41. doi: 10.1097/SLA.0000000000005928 (PMC10631507; doi:10.1097/SLA.0000000000005928)
Supplement: Supplementary file 2 [file sla-278-e1232-s002.docx]

## Supplemental Material 2. Feasibility learning curve and impact on outcomes

The primary and secondary outcomes, did not change significantly: Conversion from 8.7% to 6.2%, *P* = 0.238; major complications from 39.0% to 36.4%, *P* = 0.609; and textbook outcome from 64.8% and 69.1%, *P* = 0.387. The rate of blood loss >1000ml decreased from 9.6% to 4.0%, *P* = 0.006. The CUSUM analysis of conversion and blood loos revealed a turning point at 25, and 42 RPD procedures, respectively. After #N = 37, conversion decreased from 10.8% versus 5.0%, *P* = 0.009. After #N = 43, blood loss decreased from median 250 cc [100-500] to median 200 cc [100-400], *P* = 0.005, of which 9.6% versus 4.0% 1000 cc or more, *P* = 0.006.

| **Supplementary Table. Postoperative Outcomes in Learning Phases** | | | |
| --- | --- | --- | --- |
|  | **Before Feasibility**  **cut-off ≤ 15**  **(n = 105)** | **After Feasibility cut-off > 15**  **(n = 530)** | ***p-value*** |
| **Postoperative outcomes** |  |  |  |
| **Length of initial hospital stay, median days (IQR)** | **13 [9-21]** | **11 [7-19]** | **0.029** |
| **Initial hospital stay < 7 days, n (%)** | **7 (6.7)** | **91 (17.2)** | **0.002** |
| **Readmission, n (%)** | **28 (26.7)** | **117 (22.1)** | **0.315** |
| **Clavien-Dindo complication ≥ III, n (%)** | **41 (39.0)** | **193 (36.4)** | **0.609** |
| **Postoperative pancreatic fistula (B/C), n (%)** | **19 (18.1)** | **152 (28.7)** | **0.026** |
| **Postoperative pancreatic fistula grade C, n (%)** | **4 (3.8)** | **7 (1.3)** | **0.074** |
| **Bile leakage (B/C), n (%)** | **12 (11.2)** | **39 (7.5)** | **0.018** |
| **Delayed gastric emptying (B/C), n (%)** | **39 (37.1)** | **110 (20.8)** | **<0.001** |
| **Postpancreatectomy hemorrhage (B/C), n (%)** | **12 (11.4)** | **65 (12.3)** | **0.396** |
| **Chyle leakage (Grade B/C), n (%)** | **2 (1.9)** | **(2.8)** | **0.886** |
| **Wound infection, n (%)** | **11 (10.5)** | **29 (5.5)** | **0.056** |
| **Oncologic outcomes** |  |  |  |
| **Tumor size, mm [IQR]** | **23 [14-32]** | **25 [18-35]** | **0.058** |
| **Lymph node harvest^, n [IQR]** | **15 [12-19]** | **15 [11-19]** | **0.058** |
| R0 resection^**, n (%)** | **25/89 (71.9)** | **332/451 (73.8)** | **0.848** |
| **Mortality** |  |  |  |
| **In-hospital/30-day mortality, n (%)** | **2 (1.9)** | **20 (3.8)** | **0.385** |


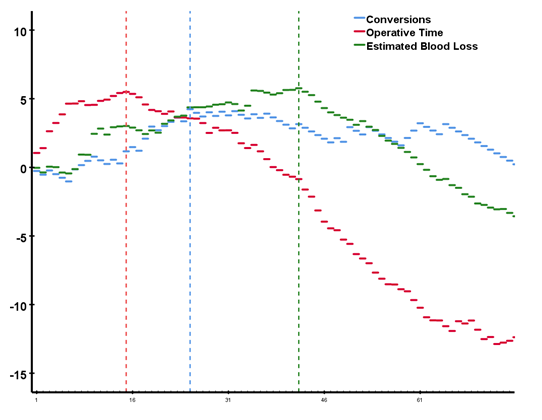


**FIGURE 1. Learning Curves for Conversion, Operative Time, and Estimated Blood Loss of Robotic Pancreatoduodenectomy**
