# Supplementary material for: The Feasibility, Proficiency, and Mastery Learning Curves in 635 Robotic Pancreatoduodenectomies Following a Multicenter Training Program: “Standing on the Shoulders of Giants”
Source: Ann Surg. 2023 Jun 8;278(6):e1232–41. doi: 10.1097/SLA.0000000000005928 (PMC10631507; doi:10.1097/SLA.0000000000005928)
Supplement: Supplementary file 3 [file sla-278-e1232-s003.docx]

## Supplemental Material 3. Table: Survey Outcomes

| **Supplemental Table. Survey Outcomes** | | |
| --- | --- | --- |
|  | **n = 15** | |
| **Surgical experience** |  | |
| **Years as a practicing surgeon, years [IQR]** | **14 [10-21]** | |
| **Current elective surgical procedures performed, n (%)** |  | |
| Pancreatic | **14 (93)** | |
| **Liver** | **8 (53)** | |
| **Colorectal** | **5 (33)** | |
| **Upper-GI** | **1 (7)** | |
| **Bariatric** | **3 (20)** | |
| **Years of experience with advanced* MI gastrointestinal resections, years [IQR]** | **15 [10-24]** | |
| **Type of Ml gastrointestinal surgery do you performed, n (%)** |  | |
| **Pancreatic** | **15 (100)** | |
| **Liver** | **8 (53)** | |
| **Colorectal** | **7 (47)** | |
| **Upper-GI** | **2 (13)** | |
| **Bariatric** | **2 (13)** | |
| **Type of MIS-HPB surgery performed for more than 1 year prior to participating in LAELAPS-3, n (%)** |  | |
| **None** | **0 (0)** | |
| **Laparoscopic distal pancreatectomy** | **4 (27)** | |
| **Robotic distal pancreatectomy** | **8 (53)** | |
| **Laparoscopic pancreatoduodenectomy** | **4 (27)** | |
| **Laparoscopic minor liver resection** | **8 (53)** | |
| **Robotic minor liver resection** | **5 (33)** | |
| **Laparoscopic major liver resection** | **2 (13)** | |
| **Robotic major liver resection** | **0 (0)** | |
| **Volumes and team** |  | |
| **Minimum required center volume of RPD, median [IQR]** | **19 [10-25]** | |
| **Minimum required surgeon volume of RPD, median [IQR]** | **14 [10-20]** | |
| **Own center’s volume** | **20 [13-25]** | |
| **Individual volume in past 3 years** | **45 [30-60]** | |
| **Number of surgeons performing RPDs per center** | **3 [2-3]** | |
| **Surgical team, n (%)** |  | |
| **Two LAELAPS-3 surgeons** | **12 (80)** | |
| **One LAELAPS-3 surgeon and one other surgeon** | **2 (13)** | |
| **One LAELAPS-3 surgeon** | **1 (7)** | |
| **Case selection** |  | |
| Main (relative) contra-indications**, n (%)** | Initial 20 RPD | Current |
| **Any venous abutment** | **(73)** | **10 (67)** |
| **>180° venous abutment** | **1 (7)** | **3 (20)** |
| **Any arterial abutment** | **11 (73)** | **13 (87)** |
| **>180° arterial abutment** | **1 (7)** | **3 (20)** |
| Suspected/confirmed malignancy | **1 (7)** | **0 (0)** |
| Previous complicated upper abdominal surgery | **4 (27)** | **3 (20)** |
| Requiring additional colonic/mesocolon resection | **7 (47)** | **4 (27)** |
| BMI > 30 | **1 (7)** | **0 (0)** |
| BMI > 35 | **4 (27)** | **2 (13)** |
| Chronic pancreatitis | **7 (47)** | **3 (20)** |
| Recurrent acute pancreatitis | **5 (33)** | **5 (33)** |
| Post-ERCP pancreatitis (1x) | **4 (27)** | **1 (7)** |
| Other, namely logistic problems (no access to robot) | **3 (20)** | **1 (7)** |
| **Training** |  | |
| Times proctored in a RPD procedure, mean (SD) | **5 (3)** | |
| **Type of pancreatic surgery training did you performed** |  | |
| **Simulation (da Vinci skills simulator)** | **15 (100)** | |
| Video |  | |
| 1-5 hours | **7 (47)** | |
| 5-10 hours | **7 (47)** | |
| 20-30 hours | **0 (0)** | |
| >40 hours | **1 (7)** | |
| Artificial organs |  | |
| Pittsburgh protocol (LAELAPS-3/E-MIPS course/LEARNBOT) | **11 (73)** | |
| Outside Pittsburgh protocol | **(18)** | |
| Case observation | **15 (100)** | |
| Proctoring |  | |
| Off-site | **11 (73)** | |
| On-site | **15 (100)** | |
| Fellowship | **(18)** | |
| **Experience** |  | |
| **How likely are you to recommend the LAELAPS-3 program? Median [IQR]** | **9 [8.5-10]** | |
| **Minimum volume of required for RPD** |  | |
| **Center** | **19 ± 11** | |
| **Surgeon** | **14 ± 5** | |
| **Top 3 valuable technical aspects (details) learned from LEALAPS-3** |  | |
| 1. **Structured methods for the anastomosis** | **11 (73)** | |
| 1. **Strict adherence to procedural steps** | **8 (53)** | |
| 1. Value of feedback by peers | **5 (33)** | |
| Most prevalent changes after fully trained in LAELAPS-3 |  | |
| 1. Interrupted sutures for the HJ | **8 (53)** | |
| 1. Mobilization of first jejunal loop from the right side | **4 (27)** | |
| 1. Gastric anastomose via open incision (to gain time) | **4 (27)** | |
| *Beyond appendectomy, cholecystectomy | | |
