# Supplementary material for: The Feasibility, Proficiency, and Mastery Learning Curves in 635 Robotic Pancreatoduodenectomies Following a Multicenter Training Program: “Standing on the Shoulders of Giants”
Source: Ann Surg. 2023 Jun 8;278(6):e1232–41. doi: 10.1097/SLA.0000000000005928 (PMC10631507; doi:10.1097/SLA.0000000000005928)
Supplement: Supplementary file 4 [file sla-278-e1232-s004.docx]

## Supplemental Material 4. Figure: Proficiency and Mastery Learning Curves of Textbook Outcome in Subgroups with and without Laparoscopic Pancreatoduodenectomy Experience


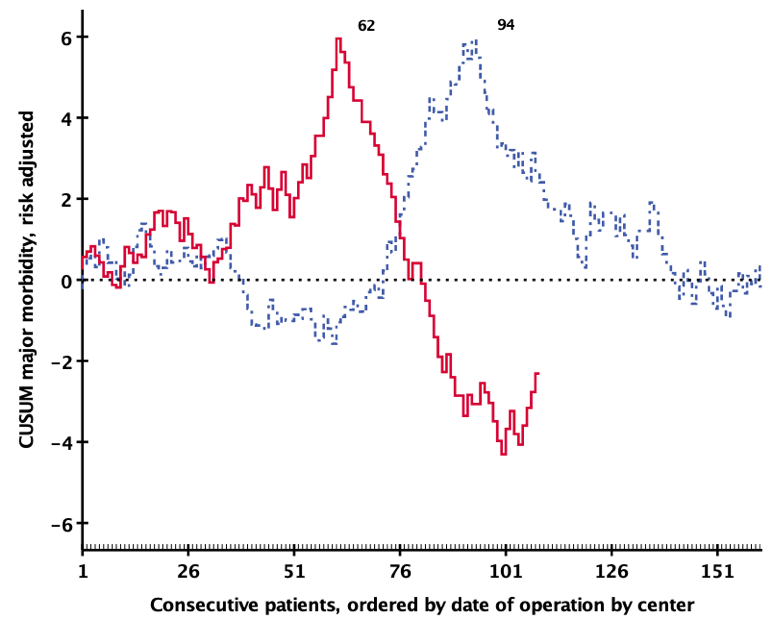

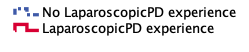

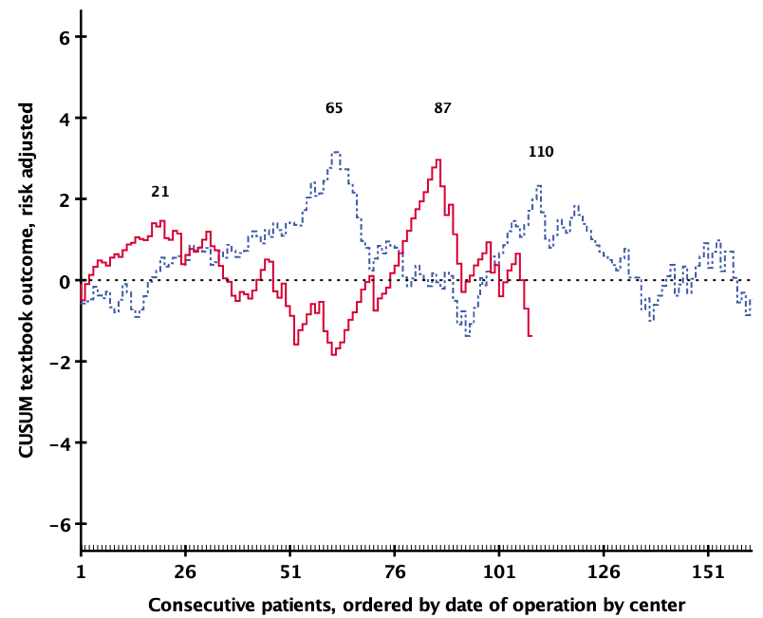

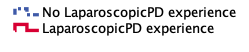


**Legend**: The X-axis indicates groups of consecutive RPDs ranked from first to last per center, and the lines indicates the risk-adjusted cumulative sum analysis for proficiency (left) and mastery (right). The red colored line indicated the learning curve for centers with laparoscopic pancreatoduodenectomy experience. The blue colored line indicated the learning curve for centers without laparoscopic pancreatoduodenectomy experience
