# Supplementary material for: The Feasibility, Proficiency, and Mastery Learning Curves in 635 Robotic Pancreatoduodenectomies Following a Multicenter Training Program: “Standing on the Shoulders of Giants”
Source: Ann Surg. 2023 Jun 8;278(6):e1232–41. doi: 10.1097/SLA.0000000000005928 (PMC10631507; doi:10.1097/SLA.0000000000005928)
Supplement: Supplementary file 5 [file sla-278-e1232-s005.docx]

## Supplemental Material 5. Outcomes based on Miami guidelines annual volume criteria

| **Supplemental Table. Postoperative Outcomes in Volume Groups** | | | |
| --- | --- | --- | --- |
|  | **<20/year** | **≥20/year** | ***p =*** |
| **Centers volume outcomes** | **n = 141** | **n = 494** |  |
| **Operative time, min, median [IQR]** | **405 [342-468]** | **395 [341-465]** | **.982** |
| **Blood loss, mL, median [IQR]** | **211 [100-500]** | **200 [100-400]** | **.252** |
| **Conversion, n (%)** | **16 (11.4)** | **26 (5.3)** | **.011** |
| **Clavien-Dindo complication ≥ III, n (%)** | **46 (32.6)** | **188 (38.1)** | **.238** |
| **In-hospital/30-day mortality, n (%)** | **8 (5.7)** | **14 (2.8)** | **.085** |
| **Surgeon volume outcomes** | **n = 230** | **n = 405** |  |
| **Operative time, min, median [IQR]** | **383 [340-458]** | **400 [341-470]** | **.128** |
| **Blood loss, mL, median [IQR]** | **250 [100-500]** | **200 [100-400]** | **.034** |
| **Conversion, n (%)** | **22 (9.6)** | **20 (4.9)** | **.024** |
| **Clavien-Dindo complication ≥ III, n (%)** | **75 (32.6)** | **159 (39.3)** | **.095** |
| **In-hospital/30-day mortality, n (%)** | **7 (3.0)** | **15 (3.7)** | **.662** |

**Supplemental Figure. Cumulative Number of Robotic Pancreatoduodenectomies Per Center over the Study Period.**


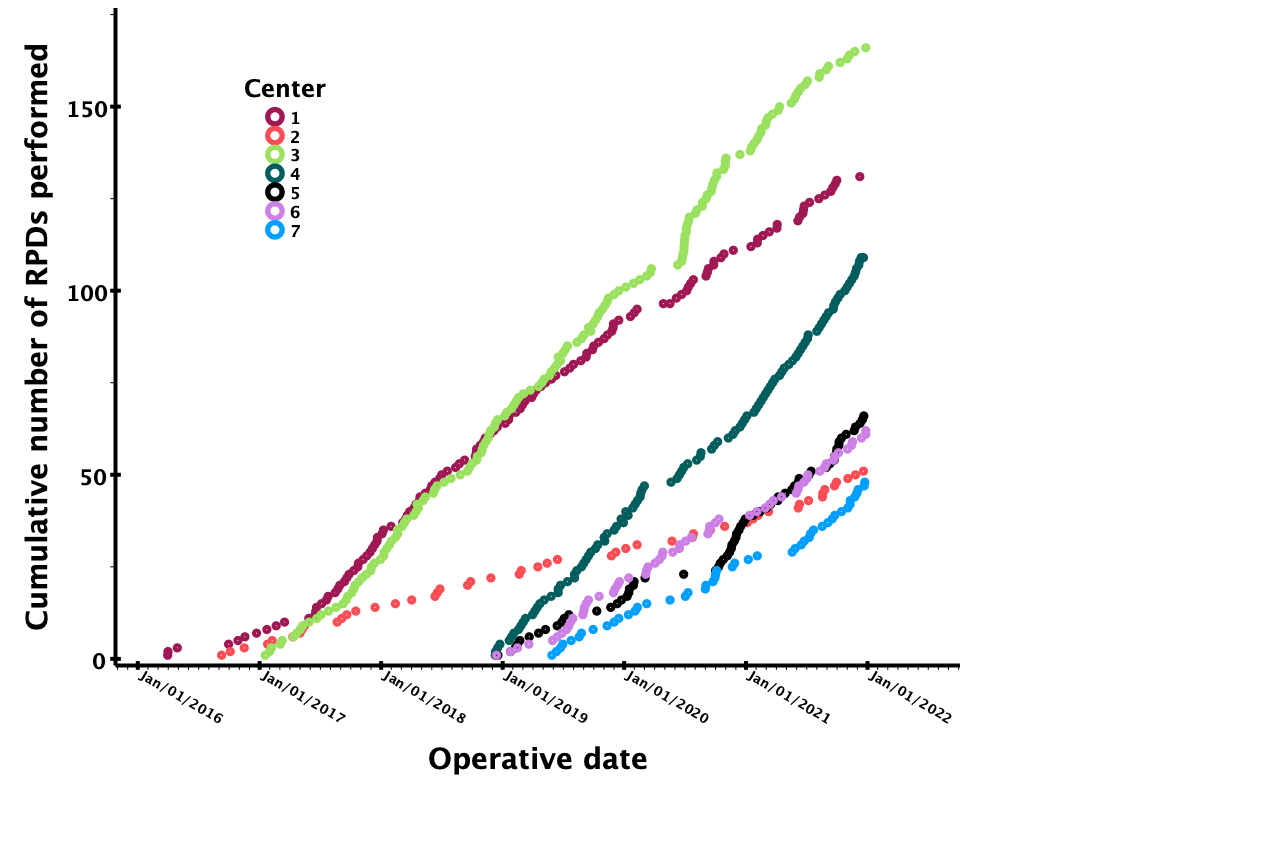


**Legend**: The X-axis indicates operative date; the Y-axis indicate the cumulative number of RPDs performed per center. Colors indicate the center.
