# Supplementary material for: The Feasibility, Proficiency, and Mastery Learning Curves in 635 Robotic Pancreatoduodenectomies Following a Multicenter Training Program: “Standing on the Shoulders of Giants”
Source: Ann Surg. 2023 Jun 8;278(6):e1232–41. doi: 10.1097/SLA.0000000000005928 (PMC10631507; doi:10.1097/SLA.0000000000005928)
Supplement: Supplementary file 6 [file sla-278-e1232-s006.docx]

## Supplemental Material 6. Outcomes of the current DPCG series versus UPMC series.

| Table 4. Current multicenter series versus the UPMC series | | |  |
| --- | --- | --- | --- |
|  | **Current DPCG series**  **(7 centers)**  **n = 635** | **UPMC series**  **(1 center)**  **N = 500** | |
| **Operative** |  |  | |
| **Total operative time, min., mean (SD)** | **414 (111)** | **415 (107)** | |
| **Blood loss, mL, median [IQR]** | **200 [100-450]** | **250 [150-400]** | |
| **Conversion, n (%)** | **42 (6.6)** | **26 (5.2)** | |
| **Postoperative** |  |  | |
| **Clavien-Dindo complication ≥ III, n (%)** | **234 (36.9)** | **124 (24.8)** | |
| **Postoperative pancreatic fistula, grade B/C n (%)** | **171 (**26.9) | **39 (7.8)** | |
| **Reoperation, n (%)** | **62 (9.8)** | **25 (5.0)** | |
| **In-hospital/30-day mortality, n (%)** | **22 (3.5)** | **9 (1.8)** | |
